# Evaluation of Performance of Detection of Immunoglobulin G and Immunoglobulin M Antibody Against Spike Protein of SARS-CoV-2 by a Rapid Kit in a Real-Life Hospital Setting

**DOI:** 10.3389/fmicb.2022.802292

**Published:** 2022-04-25

**Authors:** Monica Irungbam, Anubhuti Chitkara, Vijay Kumar Singh, Subash Chandra Sonkar, Abhisek Dubey, Aastha Bansal, Ritika Shrivastava, Binita Goswami, Vikas Manchanda, Sonal Saxena, Ritu Saxena, Sandeep Garg, Farah Husain, Tanmay Talukdar, Dinesh Kumar, Bidhan Chandra Koner

**Affiliations:** ^1^Department of Biochemistry, Maulana Azad Medical College and Associated Hospitals, New Delhi, India; ^2^Multidisciplinary Research Unit (MRU), Maulana Azad Medical College and Associated Hospitals, New Delhi, India; ^3^Department of Microbiology, Maulana Azad Medical College and Associated Hospitals, New Delhi, India; ^4^Emergency Department, Lok Nayak Jai Prakash Narayan (LNJP) Hospital, New Delhi, India; ^5^Department of Medicine, Lok Nayak Jai Prakash Narayan (LNJP) Hospital, New Delhi, India; ^6^Department of Anesthesiology, Lok Nayak Jai Prakash Narayan (LNJP) Hospital, New Delhi, India; ^7^Department of TB & Chest Diseases/Pulmonary Medicine, Lady Hardinge Medical College (LHMC), New Delhi, India; ^8^Food Safety and Standards Authority of India, Ministry of Health and Family Welfare (MoHFW), New Delhi, India

**Keywords:** SARS-CoV-2, COVID-19, receptor-binding domain (RBD), spike surface glycoprotein, chemiluminescence analysis, rapid antibody tests for COVID-19

## Abstract

**Background:**

Antibody testing is often used for serosurveillance of coronavirus disease 2019 (COVID-19). Enzyme-linked immunosorbent assay and chemiluminescence-based antibody tests are quite sensitive and specific for such serological testing. Rapid antibody tests against different antigens are developed and effectively used for this purpose. However, their diagnostic efficiency, especially in real-life hospital setting, needs to be evaluated. Thus, the present study was conducted in a dedicated COVID-19 hospital in New Delhi, India, to evaluate the diagnostic efficacy of a rapid antibody kit against the receptor-binding domain (RBD) of the spike protein of severe acute respiratory syndrome coronavirus 2 (SARS-CoV-2).

**Methods:**

Sixty COVID-19 confirmed cases by reverse transcriptase–polymerase chain reaction (RT-PCR) were recruited and categorized as early, intermediate, and late cases based on the days passed after their first RT-PCR–positive test report, with 20 subjects in each category. Twenty samples from pre-COVID era and 20 RT-PCR–negative collected during the study period were taken as controls. immunoglobulin M (IgM) and immunoglobulin G (IgG) antibodies against the RBD of the spike (S) protein of SARS-CoV-2 virus were detected by rapid antibody test and compared with the total antibody against the nucleocapsid (N) antigen of SARS-CoV-2 by electrochemiluminescence-based immunoassay (ECLIA).

**Results:**

The detection of IgM against the RBD of the spike protein by rapid kit was less sensitive and less specific for the diagnosis of SARS-CoV-2 infection. However, diagnostic efficacy of IgG by rapid kit was highly sensitive and specific when compared with the total antibody against N antigen measured by ECLIA.

**Conclusion:**

It can be concluded that detection of IgM against the RBD of S protein by rapid kit is less effective, but IgG detection can be used as an effective diagnostic tool for SARS-CoV-2 infection in real-life hospital setting.

## Introduction

The world is facing the outbreak of coronavirus disease 2019 (COVID-19), which has become a public health event of international concern (Afzal, 2020; Dubey et al., 2020, 2021; Feng et al., 2020; He et al., 2020; Nilsson et al., 2021). Accurate and rapid diagnosis of severe acute respiratory syndrome coronavirus 2 (SARS-CoV-2) infection is needed for prompt and effective patient care. Quantitative reverse transcriptase–polymerase chain reaction (RT-PCR) is the clinically accepted standard method for molecular diagnosis of SARS-CoV-2 detection. Alternatively, rapid antigen test (RAT) kit is also used for COVID-19 diagnosis. However, RT-PCR test has 70% sensitivity and 95% specificity and poses risks related to specimen collection and sample handling (Long et al., 2020a; Pray et al., 2021). A recent meta-analysis revealed that the average sensitivity and specificity of RAT for SARS-CoV-2 were 56.2 and 99.5%, respectively (Long et al., 2020b). These tests may also be falsely negative due to quality of sample or timing of carrying the test as the viral load in upper respiratory tract secretions peaks in the first week of symptoms, but may decline below the limit of detection in those presenting later. In individuals who have recovered, RT-PCR provides no information about prior exposure or immunity. Addressing this concern, various researchers developed a solution to minimize these risks by assaying immunoglobulin G (IgG) and immunoglobulin M (IgM) against the virus. Serological testing with good detection performance can be used as supplementary diagnosis of COVID-19 suspect cases with negative nucleic acid test result (Young et al., 2020). Also, to diagnose patients after the acute phase of the infection or with atypical clinical presentation with no nasopharyngeal shedding of the virus, serology testing is very useful (Noh et al., 2020; Young et al., 2020; Alsaud et al., 2021). In addition, serological testing provides a useful surveillance tool to track seroprevalence and assess the immune status in a community and may also be useful for decisions on lockdown entry–exit strategies (Parai et al., 2021).

The human body produces specific antibodies after the virus invades. The specific IgM antibody appears first, and then the titer of IgG antibody rises. Thus, the detection of IgM and IgG is believed to be another important diagnostic tool along with RT-PCR and RAT. The tests available detects anti–SARS-CoV-2 immunoglobulins, which are usually formed in the patient’s body at the earliest by 1 week and on average within 2–3 weeks from the onset of infection (Jacofsky et al., 2020; Long et al., 2020a).

Various SARS-CoV-2 serological tests using different targeted antigenic proteins have been available now. Some of them use whole virus lysate, recombinant full S (spike) or N (nucleocapsid) proteins, peptides of the N, or specific domains S1, S2, or RBD (receptor-binding domain) of the S protein. Studies have shown that S and N proteins of the virus are the most immunogenic, and these serological tests can be performed with various techniques (Saif, 1993; Duan et al., 2020). Enzyme-linked immunosorbent assay (ELISA) and chemiluminescence are considered standard methods for the same. Rapid antibody kits are also available as a point-of-care testing tool. This rapid serological test is a simple procedure, needs no special equipment, is relatively cheap, and gives fast results. However, utility of this type of rapid antibody detection kit in the diagnosis of COVID-19 in real-life hospital settings is warranted. This study is conducted to evaluate the sensitivity and specificity of a rapid antibody kit in real-life hospital setting that detects IgM and IgG separately by comparing with the total antibody detection by chemiluminescence method.

## Materials and Methods

The observational study was conducted in the Department of Biochemistry in collaboration with the Multidisciplinary Research Unit, Maulana Azad Medical College and Department of Medicine Lok Nayak Hospital, New Delhi, India, after ethical approval. Ethical approval number of the study is F.1/IEC/MAMC/(81/09/2020/No: 278) dated 24 November 2020. The study was carried out between December 2020 and June 2021.

### Cases and Controls

All the cases included in this study were COVID-19 cases confirmed by RT-PCR. Blood samples sent in red-capped blood collection vials for other biochemical tests were used, and no separate sample from them was collected for the study. Patients were categorized as “early cases” (group I) if they were recruited within the first week of positive RT-PCR test, as “intermediate cases” (group II) if taken between 8 and 14 days, and “late cases” (group III) if recruited after 14 days of their first RT-PCR–positive test. Recruitment continued until 20 samples were collected in each group. Blood sample was collected from 20 RT-PCR–negative subjects who were treated as controls. Twenty serum samples collected during January 2019 to June 2019 and preserved at −80°C were used as pre–COVID-19 era controls. Pre–COVID-19 samples (archived serum sample of pre–COVID-19) was used as negative controls for additional confirmation of test results.

### Reference Method of Anti–COVID-19 Antibody Assay

Total antibody against the nucleocapsid (N) antigen of SARS-CoV-2 was assayed from the serum of these samples using electrochemiluminescence (ECL)–based Elecsys^®^ Anti–SARS-CoV-2 immunoassay kit manufactured by Roche Diagnostics adapted to fully automated Cobas e411 immunoassay system. The cut-off indices for positive immunoassay was greater than 1.0 (arbitrary unit) by this method.

### Test Procedure for Antibody Detection Method by Rapid Kit

The ImmunoQuick COVID-19 kit evaluated in this study was produced by ImmunoScience India Private Limited, Pune, Maharashtra, India. ImmunoQuick COVID-19 was developed using the principle of immunochromatography for the rapid and qualitative detection of IgM and IgG antibodies against the RBD of spike (S) protein of COVID-19 virus in human serum, plasma, or whole blood. In an internal evaluation of the performance characteristics of the rapid kit carried out by the manufacturer, serum, plasma, and whole blood samples of total 125 RT-PCR–negative and 160 RT-PCR COVID-19–positive were used. Sensitivity and specificity were found to be 97.5% (156/160) and 98.4% (123/125), respectively. Cross-reactivity studied with dengue-, HIV-, hepatitis C virus-, and HBsAg-positive serum samples showed no cross-reactivity. Endpoint titer was found satisfactory. External evaluation of the ImmunoQuick COVID-19 IgM/IgG test kit was performed by the National Institute of Virology–Indian Council of Medical Research, Department of Health Research, Government of India at Pune and was found satisfactory as per the Centers for Disease Control and Prevention and World Health Organization guidelines. The manufacturer obtained the regulatory approval for manufacturing the kit from the Central Drugs Standard Control Organization under Directorate General of Health Services, Ministry of Health & Family Welfare, Government of India. The manufacturing of the kit followed the ISO 13485:2016 QMS guidelines. The tests were performed at the Immunoassay Laboratory of Department of Biochemistry, Maulana Azad Medical College, New Delhi, by laboratory technicians/resident doctors immediately after receiving the blood samples by the method described below:

Test cassette, specimens, buffer, and/or controls were allowed to equilibrate to the temperature of an air-conditioned room (20–22°C) prior to testing. The test cassette was removed from the sealed foil pouch and was used as earliest possible. At the time of executing the test, it was made sure that the test device was placed on a clean and level surface. Sample was taken with mini plastic dropper provided in the kit, and one drop of it was transferred to the specimen well (marked as S on the cassette) of the test device. Then two drops of sample buffer were added to the buffer well (B) immediately, and air bubbles were avoided while dispensing it. As the fluid migrates through the membrane on which antigens are impregnated, the color line(s) develop. The results were read within 10 min. The results were visible as soon as 2 min. The result was read as negative when the colored line in the control line region (C) changed from blue to red and no line appeared in the test line regions M or G. The test result indicated the presence of IgM anti–SARS-CoV-2 antibodies when the colored line in the control line region (C) changed from blue to red and a colored line appeared in the test line region M. The test result indicated the presence of IgG anti–SARS-CoV-2 antibodies when the colored line in the control line region (C) changed from blue to red and a colored line appeared in the test line region G. The test results indicated the presence of IgM and IgG anti–SARS-CoV-2 antibodies when the colored line in the control line region (C) changed from blue to red and two colored lines appear in the test line regions M and G. The result was considered as invalid when the control line was partially red and failed to completely change from blue to red. Insufficient specimen volume or incorrect procedural techniques were the most likely reasons for control line failure. During the study, we never encountered any invalid test result.

### Statistical Analysis

Data of the test results were arranged in the table as follows:

**Table:** 

	ECL anti–SARS-CoV-2–positive	ECL anti–SARS -CoV-2–negative
Rapid kit antibody–positive	a	b
Rapid kit antibody–negative	c	d

Specificity, sensitivity, negative predictive value (NPV), and positive predictive value (PPV) of anti-RBD IgM and IgG were calculated from a 2 × 2 table using formulas as follows:


Sensitivity=[a/(a+c)]×100

Specificity=[d/(b+d)]×100

PPV=[a/(a+b)]×100

NPV=[d/(c+d)]×100

The strength of the agreement of the two methods was calculated by using the Cohen κ index. Results were interpreted according to the following κ values: (i) 0.01–0.20, slight agreement; (ii) 0.21–0.40, fair agreement; (iii) 0.41–0.60, moderate agreement; (iv) 0.61–0.80, substantial agreement; and (v) 0.81–1.00, perfect agreement. *p* < 0.05 was considered statistically significant for all statistical tests.

## Results

Of the 60 patients enrolled in the study, patients were having mild, moderate, or severe COVID-19 and were divided into three groups as early, intermediate, and late cases as previously mentioned. Male-to-female ratio was 14:6 in group I, 16:4 in group II, and 15:5 in group III. Gender difference in these groups was statistically insignificant. The ages of the participants in the study groups ranged from 21 to 68 years, with no statistically significant difference among the groups. Of 100 participants, 16% were smokers, and 10% were smokers and alcoholic as well. The distribution of comorbid conditions in different groups is shown in Table 1.

**TABLE 1:** Distribution of various comorbidities among different study groups.

Comorbidity	0–7 days (group I) (*n* = 20)	8–14 days (group II) (*n* = 20)	>14 days (group III) (*n* = 20)	PCR-negative and pre-COVID era group (*n* = 40)
Diabetes mellitus (DM)	4	2	2	0
Hypertension (HTN)	2	1	3	0
DM + HTN	6	5	0	0
Chronic pulmonary obstructive disease (COPD)	1	1	0	0
DM + COPD + renal failure	1	1	0	0
Coronary artery disease (CAD)	–	1	–	–
DM + CAD	1	1	–	–
Bronchial asthma + HTN		–	2	–
Spondylitis		–	1	–
No comorbidity	5	8	12	40
Smokers	5	4	5	2
Smoker and alcoholic as well	3	4	2	1

The results of total antibody positive (level >1.0) and negative (level <1.0) against N protein of SARS-CoV-2 virus and achieved pre-COVID era non-COVID samples by ECL-based immunoassay (ECLIA) are presented in Table 2.

**TABLE 2:** Distribution of total antibody against nucleocapsid protein of SARS-CoV-2 test results at 0–7, 8–14, and >14 days of RT-PCR–positive confirmed COVID-19 cases, PCR-negative subjects, and pre–COVID-19 controls.

Antibody titer against nucleocapsid protein by ECLIA	COVID cases confirmed by RT-PCR			PCR–negative subjects	Pre-COVID samples	Fisher exact test (*p*)
	0–7 days (group I)	8–14 days (group II)	>14 days (group III)			
>1.0	16	19	20	2	0	<0.00001
<1.0	4	1	0	18	20	

*p-value by Fischer exact test for this 5 × 2 table was <0.00001, which is statistically significant.*

## Discussion

The present study was designed to evaluate the diagnostic efficacy of a rapid antibody kit against the RBD of the S protein of COVID-19 in a real-life hospital setting. Among the diagnostic immunoassay methods, chemiluminescence-based immunoassay (CLIA) is considered to be very sensitive and effective. In the present study, diagnostic efficacy of total antibody assay against nucleocapsid protein by ECL method in the early, intermediate, and late cases was performed. Tables 2, 3 show that during first 7 days after PCR positivity, the sensitivity, specificity, PPV, and NPV of ECLIA-based antibody assay were 80, 100, 100, and 83.3%, respectively; between 8 and 14 days after PCR positivity, those were 95, 100, 95, and 95.2%, respectively, and for late cases, those were 100% for all indices. This indicates that ECLIA is a very effective diagnostic tool for COVID-19, and its efficacy after 7 days is very close to that of RT-PCR. Even within 7 days, it was found to be very effective. Thus, for comparison of any other antibody testing method for COVID-19, ECLIA-based immunoassay of total antibody against nucleocapsid protein can be considered as a standard method.

**TABLE 3:** Accuracy indices of total antibody assay against nucleocapsid protein by ECLIA at 0–7, 8–14, and >14 days of COVID diagnosis by RT-PCR with reference to pre-COVID era samples and RT-PCR–negative samples collected during the study period.

	Within 0–7 days of RT-PCR report	Within 8–14 days of RT-PCR report	>14 days of RT-PCR report
**With reference to pre-COVID era samples**			
Sensitivity (%)	80	95	100
Specificity (%)	100	100	100
PPV (%)	100	95	100
NPV (%)	83.3	95.2	100
**With reference to RT-PCR–negative samples collected during study period**			
Sensitivity (%)	80	95	100
Specificity (%)	80	80	80
PPV (%)	88	90.4	90.9
NPV (%)	66.6	88.8	100

For the assessment of seroprevalence, most of the authorities recommend antibody assay either by ELISA or CLIA-based methods. The manufacturers of rapid antibody assays claim that rapid test is also effective in serosurveillance particularly in remote areas where there are no laboratory facilities. However, the diagnostic efficacy of these rapid antibody kit has also been evaluated in standard laboratory conditions (Li et al., 2020; Zhang et al., 2020), but not in real-life hospital settings treating COVID-19 patients. Performance evaluation in real-life situation is an important component of postmarket surveillance. There are limited data to prove their efficacy in the hospital setting. Thus, in this study, we tried that in a dedicated COVID hospital.

Tables 4, 5 show that the assay of IgM by rapid kit has sensitivity of 37.5% for early cases and 31.6% for intermediate cases, although specificity and PPV were 100%. NPV was found to be 28 and 7.14%, and Cohen κ was 0.1935 and 0.044, respectively. After 14 days, as expected, IgM antibody by rapid kit was undetectable because of class switching of the antibody. Hence, we conclude that IgM detection by rapid kit has very limited effectiveness as a diagnostic tool for COVID-19 in real-life hospital setting.

**TABLE 4:** Table showing distribution of positive and negative antibody test results by rapid test kit and ECLIA method on samples collected from COVID patients at 0–7, 8–14, and after 14 days of RT-PCR–positive and RT-PCR–negative subjects and pre-COVID samples.

			Total antibody assayed by ECL method									
			Within 0–7 days of RT-PCR		Within 8–14 days of RT-PCR		After 14 days of RT-PCR		RT-PCR–negative		Pre-COVID era samples	
Rapid test			Positive	Negative	Positive	Negative	Positive	Negative	Positive	Negative	Positive	Negative
	IgM	Positive	06	00	06	00	00	00	01	00	00	00
		Negative	10	04	13	01	20	00	00	19	00	20
	IgG	Positive	14	01	18	00	17	00	02	00	00	00
		Negative	02	03	01	01	02	01	00	18	00	20
												

**TABLE 5:** Accuracy indices of IgM and IgG antibody (against the RBD of the spike protein of SARS-CoV-2 virus) by rapid test for the diagnosis of COVID-19 (calculated by taking total antibody levels by ECL as standard methods.

	Diagnostic accuracy of IgM by rapid kit					Diagnostic accuracy of IgG by rapid kit				
	Sample collected within 0–7 days of positive RT-PCR	Sample collected within 8–14 days of positive RT-PCR	Sample collected after 14 days of positive RT-PCR	Pre-COVID samples	RT-PCR–negative	Sample collected within 0–7 days of positive RT-PCR	Sample collected within 8–14 days of positive RT-PCR	Sample collected with after 14 days of positive RT-PCR	Pre-COVID samples	RT-PCR –negative
Sensitivity (%)	37.5	31.6	0	100	100	87.5	94.73	89.47	100	100
Specificity (%)	100	100	0	100	100	75	100	100	100	100
PPV (%)	100	100	0	100	100	93	100	100	100	100
NPV (%)	28	7.14	0	100	100	60	50	33.3	100	100
Cohen κ	0.1935	0.044	0			0.5714	0.6428	0.459		

As shown in Tables 4, 5, detection of IgG by rapid kit for early intermediate and late cases was 87.5, 94.7, and 89.5%, respectively, and specificity was 75, 100, and 100%, respectively. Similarly, PPV was 93, 100, and 100%, respectively; NPV was 60, 50, and 33.3%, respectively; and Cohen κ was 0.5714, 0.6428, and 0.459, respectively. This indicates that IgG detection is effective enough in the diagnosis of COVID-19 in the hospital setting. Even within 0–7 days, its sensitivity and specificity were high, indicating a possible early class switching of antibody against the RBD of the spike protein. These observations go against the contention that antibody detection or assay best suits the serosurveillance and not the diagnosis. From this, we conclude that the efficacy of IgG against the RBD of the spike protein is as effective as that by CLIA and can be utilized in the hospital setting. However, combining IgM detection with IgG does not improve the diagnostic efficacy and hence is a mere wastage of resources. Thus, we recommend IgG (against the RBD of S protein) assay by rapid kit in the diagnosis of COVID-19 for the screening of suspected patients where RT-PCR or CLIA-based antibody assay facility is not available. Even a variation in performances of assays is likely to exist when diagnosing different populations such as individuals with different diet habits, mental well-being, and so on (Soni et al., 2020a,b, c; Mehta et al., 2021).

However, the limitation of the present study is that while evaluating rapid test, we evaluated the detection of antibody against the RBD of the spike protein of SARS-CoV-2 virus but took assay of total antibody against a different one, that is, nucleocapsid (N) protein, as our standard reference method. Another limitation is that the study was conducted in only one hospital of New Delhi involving a limited number of samples from the local population. Immune response being variable in different populations, its applicability in other populations is worth evaluating.

## Data Availability Statement

The original contributions presented in the study are included in the article. Further inquiries can be directed to the corresponding author/s.

## Author Contributions

BK, BG, AC, SCS, SG, FH, SS, and VM conceived and designed the experiments. MI, VS, AB, AD, SCS, and RSh carried out the experiments. MI, VS, AB, and AD contributed to collection of clinical samples. AC, SCS, BG, and BK performed the analysis and analyzed the data. MI, AC, SCS, BG, and BK wrote and finalized the manuscript. BK, DK, and TT contributed to resources. All authors read and approved the final manuscript.

## Conflict of Interest

The authors declare that the research was conducted in the absence of any commercial or financial relationships that could be construed as a potential conflict of interest.

## Publisher’s Note

All claims expressed in this article are solely those of the authors and do not necessarily represent those of their affiliated organizations, or those of the publisher, the editors and the reviewers. Any product that may be evaluated in this article, or claim that may be made by its manufacturer, is not guaranteed or endorsed by the publisher.
